# Reshaping *in vitro* Models of Breast Tissue: Integration of Stromal and Parenchymal Compartments in 3D Printed Hydrogels

**DOI:** 10.3389/fbioe.2020.00494

**Published:** 2020-06-11

**Authors:** Patrícia Barros da Silva, Mariana Coelho, Sílvia Joana Bidarra, Sara Carvalheira Neves, Cristina Carvalho Barrias

**Affiliations:** ^1^i3S—Instituto de Inovação e Investigação em Saúde, Porto, Portugal; ^2^INEB—Instituto de Engenharia Biomédica, Universidade do Porto, Porto, Portugal; ^3^FEUP—Faculdade de Engenharia, Universidade do Porto, Porto, Portugal; ^4^ICBAS—Instituto de Ciências Biomédicas Abel Salazar, Universidade do Porto, Porto, Portugal

**Keywords:** 3D model, hydrogel, 3D printing, stroma, parenchyma, epithelial morphogenesis, tissue engineering

## Abstract

Breast tissue consists of an epithelial parenchyma embedded in stroma, of heterogeneous and complex composition, undergoing several morphological and functional alterations throughout females' lifespan. Improved knowledge on the crosstalk between parenchymal and stromal mammary cells should provide important insights on breast tissue dynamics, both under healthy and diseased states. Here, we describe an advanced 3D *in vitro* model of breast tissue, combining multiple components, namely stromal cells and their extracellular matrix (ECM), as well as parenchymal epithelial cells, in a hybrid system. To build the model, porous scaffolds were produced by extrusion 3D printing of peptide-modified alginate hydrogels, and then populated with human mammary fibroblasts. Seeded fibroblasts were able to adhere, spread and produce endogenous ECM, providing adequate coverage of the scaffold surface, without obstructing the pores. On a second stage, a peptide-modified alginate pre-gel laden with mammary gland epithelial cells was used to fill the scaffold's pores, forming a hydrogel *in situ* by ionic crosslinking. Throughout time, epithelial cells formed prototypical mammary acini-like structures, in close proximity with fibroblasts and their ECM. This generated a heterotypic 3D model that partially recreates both stromal and parenchymal compartments of breast tissue, promoting cell-cell and cell-matrix crosstalk. Furthermore, the hybrid system could be easily dissolved for cell recovery and subsequent analysis by standard cellular/molecular assays. In particular, we show that retrieved cell populations could be discriminated by flow cytometry using cell-type specific markers. This integrative 3D model stands out as a promising *in vitro* platform for studying breast stroma-parenchyma interactions, both under physiological and pathological settings.

## Introduction

In the twenty-first century, cancer is expected to rank as the leading cause of death worldwide, and the single most important barrier to increasing life expectancy (GLOBOCAN2018) (Ferlay et al., 2019). Among females, breast cancer is the most diagnosed cancer, accounting for almost 1-in-4 cancer cases, and the leading cause of cancer-related deaths. These numbers point breast cancer as major health threat for women, calling for the urgent implementation of measures for improved prevention, diagnosis and treatment (Ferlay et al., 2019). On the other hand, risks associated with current clinical procedures for breast reconstruction, following cancer or trauma, emphasize the need of new strategies for repairing/regenerating breast tissue (Donnely et al., 2019).

Breast tissue is complex, undergoing various morphological and functional alterations throughout a woman's lifespan. It is primarily composed of mammary gland epithelial cells and a variety of stromal cell types, which interact in different ways, depending on internal and external stimuli. Improved knowledge on these interactions would enable a better understanding on the dynamic alterations of breast tissue, underlying both healthy and pathological processes. Several studies have focused on how cellular components of the niche, including fibroblasts, adipocytes, endothelial cells and infiltrating immune cells, regulate breast epithelial cells behavior. While the different cell types act cooperatively, fibroblasts are the predominant stromal cell type, playing a key role in the regulation of morphogenesis and proliferation of normal and tumorigenic epithelial cells, being thus considered critical for tumor progression (Weigelt and Bissell, 2008; Arendt et al., 2010; Oskarsson, 2013). More recently, the importance of non-cellular components, namely the extracellular matrix (ECM) has been also emphasized (Weigelt and Bissell, 2008; Arendt et al., 2010; Oskarsson, 2013).

In cancer pathogenesis, the role of tissue stroma has become an area of intense investigation due to the mounting evidence that it can provide both tumor-suppressing and tumor-promoting environments, thus regulating epithelial neoplastic growth (Yuan et al., 2016). Therefore, understanding the crosstalk and feedback mechanisms between stromal and parenchymal compartments of breast tissue is of major relevance. Still, adequate customizable models to study these complex interactions are still lacking. *In vitro* studies using traditional 2D models have provided important insights into relevant pathophysiological processes occurring in breast tissue, and associated mechanisms (Kozlowski et al., 2009; Sung et al., 2013; Jin et al., 2018; Williams et al., 2018). Still, 2D models are reductionist as they fail to recapitulate key architectural features of healthy and diseased tissues, namely by lacking three-dimensionality, forcing artificial cell polarity and failing to mimic native biomechanical properties. On the other hand, xenograft models may not be representative of human-specific conditions (Wagner, 2003; Jackson and Thomas, 2017). In this context, the paradigm shift from 2D to 3D culture is underway and rapidly progressing, as 3D models fill the gap between traditional monolayer cultures and animal models (Pampaloni et al., 2007).

Some studies have been performed using spheroid-like 3D multicellular aggregates, both with mammary epithelial monocultures (Chandrasekaran et al., 2014; Reynolds et al., 2017) and stroma-epithelial co-cultures (Li and Lu, 2011; Lazzari et al., 2018). While these systems are helpful and better replicate a tissue-like environment, as compared to monolayer cultures, they often do not support adequate epithelial morphogenesis. Also, mild cell recovery is frequently hampered by the strong cell-cell and cell-matrix interactions that are typically established in spheroids. In contrast, 3D models where cells are entrapped in a hydrogel-based 3D matrix may be a promising alternative, proving relevant tools for insightful analysis of cell-matrix interactions and morphogenetic events. M Bissel's team elegantly demonstrated the significance of such hydrogel systems, by creating a useful prototypic model of mammary gland acini, which has been used in numerous studies (Petersen et al., 1998; Lee et al., 2007). Still, while ECM-derived protein hydrogels such as collagen and Matrigel are commonly used, they present disadvantages, such as high lot-to-lot variability, intrinsic bioactivity and poorly tuneable mechanical properties (Zaman, 2013; Gill and West, 2014). Recent advances in materials science have delivered cell-instructive/responsive hydrogels, with customizable biochemical and biomechanical properties (Fischbach et al., 2007; Gill et al., 2012; Bidarra et al., 2016), and the emergence of advanced manufacturing techniques has allowed their processing into more sophisticated 3D scaffolds. Significantly, only a few of these models combine epithelial cells with fibroblasts (Krause et al., 2008; Xu and Buchsbaum, 2012; McLane and Ligon, 2016; Koledova, 2017), and the synthesis and deposition of endogenous ECM by hydrogel-entrapped fibroblasts has not been convincingly demonstrated so far.

To address this challenge, this work focused on the development of a new 3D *in vitro* model to study breast tissue dynamics. The hybrid system combines a 3D printed alginate scaffold seeded with mammary fibroblasts and their ECM (stromal compartment) and hydrogel-embedded mammary epithelial cells (parenchymal compartment). This advanced 3D model is expected to provide a unique *in vitro* platform to study the crosstalk between stromal and mammary epithelial cells, both under physiological or pathological conditions.

## Materials and Methods

### Alginate

Pharmaceutical grade sodium alginate (LF 20/40, FMC Biopolymers) was used to produce the 3D printed scaffolds, and ultrapure sodium alginate (PRONOVA UP LVG, Novamatrix, FMC Biopolymers) was used for cell embedding. The two types of alginate presented similar guluronic acid content (ca. 70%) and molecular weight (ca. 150 kDa). Covalent grafting of the oligopeptidic RGD sequence [(Glycine)4-Arginine-Glycine-Aspartic acid-Serine-Proline, Peptide International] to alginate was performed by aqueous carbodiimde chemistry as described previously (Bidarra et al., 2011; Fonseca et al., 2011). Briefly, an alginate solution at 1 wt.% in MES buffer (0.1 M 2-(N-morpholino)ethanesulfonic acid, 0.3 M NaCl, pH 6.5) was prepared and stirred overnight (ON) at room temperature (RT). Then, N-hydroxy-sulfosuccinimide (Sulfo-NHS, Pierce) and 1-ethyl-(dimethylaminopropyl)-carbodiimide (EDC, Sigma, 27.4 mg per gram of alginate) were sequentially added at a molar ratio of 1:2, followed by 100 mg of RGD peptide (Genscript) per gram of alginate. After stirring for 20 h, the reaction was quenched with hydroxylamine (Sigma) and the solution was dialyzed against deionized water for 3 days (MWCO 3500). After purification with charcoal, RGD-alginate was lyophilized and stored at −20°C until further use. The amount of grafted peptide was 50 mg peptide per gram of alginate and was quantified using the BCA Protein Assay (Pierce) as previously described in Fonseca et al. (2011). To obtain hydrogels with different RGD amounts (200 μM, 400 μM and 600 μM), RGD-modified alginate and non-modified alginate were combined at different ratios in the gel precursor solution, before crosslinking.

### Extrusion-Based 3D Printing of RGD-Alginate Hydrogel Scaffolds

**Figure 1:**
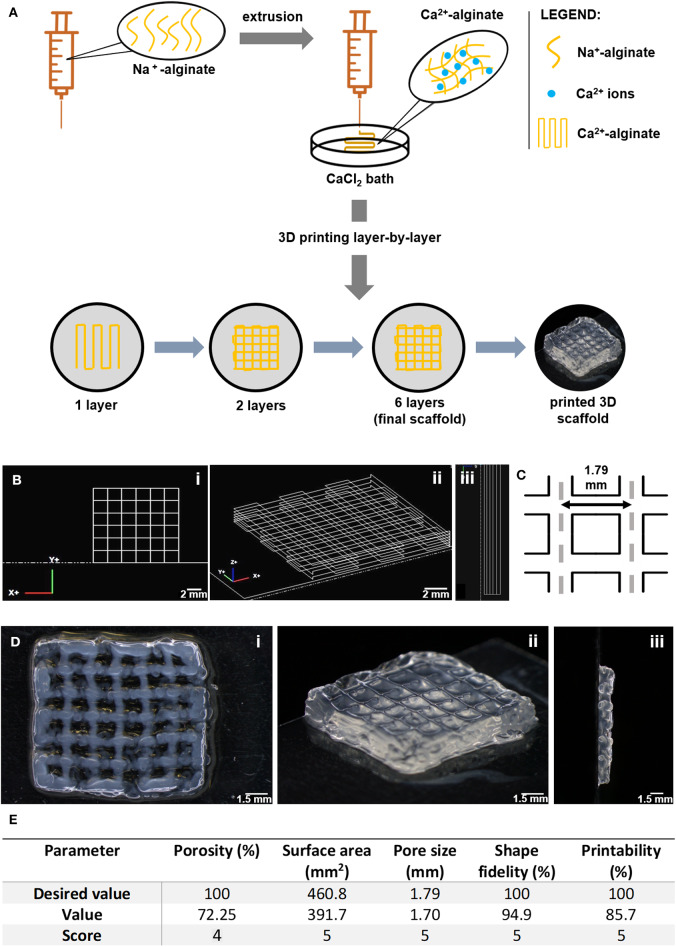
Fabrication of 3D printed porous scaffolds using RGD-alginate hydrogels. **(A)** The sodium alginate ink was extruded into a CaCl_2_ bath. The structure was printed layer-by-layer until forming a crosshatch 3D scaffold with 6 layers. **(B)** Original CAD-design of the 3D scaffolds. **(C)** Theoretical pore size and shape. **(D)** Image of 3D printed alginate scaffolds. Scale bars: 0.2 cm, 1.5 mm, 1 mm, or 0.8 mm, as per order of appearance. **(E)** All parameters are ranked on a scale from 1 to 5, in which 1 corresponds to 0–20% success in printing scaffolds, 2 corresponds to 21–40%, 3 corresponds to 41–60%, 4 corresponds to 61–80% and 5 corresponds to 81–100%.

Alginate (LF 20/40) solutions at 4 wt.% were prepared in 0.9% w/v NaCl (VWR) in distilled water and left stirring ON at RT. The extrusion-based 3D printing system was set up and the printing code was generated using NCPlot (NCPlot Software LLC) was uploaded into RepetierHost software (Hot-World GmbH & Co. KG, Germany). For printing, a sodium alginate gel-precursor solution was extruded into a 12.5 mM CaCl_2_ (VWR) crosslinking bath, using a syringe pump (equipped with a 10 mL (12 mL) luer-lock SOFT-JECT syringe with 0.210 mm nozzle diameter, at an extrusion flow of 2 mL/h, and printing speed of 170 mm/min). After layer-by-layer printing (total of 6 square-shaped layers, 0.2 mm filament width, 1.8 mm pore size), which lasted for ca. 3 min per scaffold, the 3D printed scaffold was further soaked in a 20 mM CaCl_2_ for 5 min, to consolidate crosslinking and improve stability. Afterwards, scaffolds were thoroughly washed in distilled water to remove excess calcium and stored in ethanol (70% v/v). Analysis of printed scaffolds was performed with a tereomicroscope (SZX10, Olympus Life Science Solution). For detailed scaffold analysis, image processing software ImageJ® (National Institute of Health) was used (Rueden et al., 2017). Porosity was calculated according to the following equation (Loh and Choong, 2013): Porosity (%) = (1 − V_solid_/V_total_) × 100, where: V_solid_ is calculated based on the real pore size and V_total_ is calculated based on the theoretical pore size (Figure 1). Measurements were performed on different representative scaffolds. Printing parameters were scored based on a scale from 1 to 5, in which: 1 corresponds to 0–20% of real vs. theoretical value, 2 corresponds to 21–40%, 3 corresponds to 41–60%, 4 corresponds to 61–80% and 5 corresponds to 81–100%.

### Cell Sources and Maintenance

Primary cultures of human mammary fibroblasts (hMF) from (ScienCell Research Laboratories) were routinely cultured in fibroblasts medium: high glucose Dulbecco's Modified Eagle Medium GlutaMax™ (Gibco Life Technologies) supplemented with 10% of fetal bovine serum (FBS, Gibco Life Technologies) and 1% of Penicillin-Streptomycin solution (Pen-Strep, Sigma-Aldrich). Human mammary gland epithelial cells (MCF10A, ATCC - American Type Culture Collection) were routinely cultured in epithelial cells medium: DMEM/Ham's F-12 medium (Gibco Life Technologies) supplemented with 100 ng/mL cholera toxin, 20 ng/mL epidermal growth factor, 0.01 mg/mL insulin, 500 ng/mL hydrocortisone, 5% horse serum (ThermoFisher) and 1% Pen-Strep. All growth factors were purchased from Sigma-Aldrich.

### Culture of hMF On-Top of 3D Printed Scaffolds

RGD-alginate 3D-printed scaffolds with different amounts of RGD (200, 400, and 600 μM) were placed in 24-well plates in a laminar flow hood, cut into halves with a 22 pc. scalpel (FEATHER Safety Razor Co., Ltd) and sterilized by washing 3 times in 70% v/v ethanol in dH_2_O (for 5 min each). They were then washed twice with non-supplemented medium and once with complete DMEM GlutaMax^TM^. Fibroblasts were trypsinized from T75 culture flasks, counted and seeded onto scaffolds at densities ranging from 1.0 × 10^5^ to 5.0 × 10^5^ cells *per* scaffold. Cellularized scaffolds were incubated at 37°C, and additional medium was added after 2 h. After 24–48 h, cellularized scaffolds were transferred to pHEMA-coated wells and media was changed every 1 or 2 days. To coat 24-well plates with pHEMA, a 12 mg/mL solution was prepared by dissolving pHEMA (Sigma-Aldrich) in 95% v/v ethanol. This solution was then sterilized by filtration. Wells were coated under sterile conditions at final pHEMA density of 0.8 mg/cm^2^. Culture plates were placed in the incubator to dry for 48 h at 37°C before use.

### 3D Culture of MCF10A Epithelial Cells Inside RGD-Alginate Hydrogels

For MCF10A cells entrapment, we used an *in-situ* crosslinking hydrogel formulation that had been previously optimized for another mammary epithelial cell line (Bidarra et al., 2016). MCF10A cells were trypsinized, counted and resuspended at 5 × 10^6^ cells/mL in RGD-alginate solution (UP LVG, 200 μM RGD) with crosslinking agents, and hydrogel discs were obtained as described in Bidarra et al. (2016). Briefly, a hydrogel precursor solution of 1 wt.% ultrapure sodium alginate in in 0.9 wt.% NaCl was prepared, sterile-filtered (0.22 μm) and mixed with an aqueous suspension of sterile CaCO_3_ (Fluka) at a CaCO_3_/COOH molar ratio of 1.662. Then, a fresh sterile-filtered solution of glucone delta-lactone (GDL, Sigma-Aldrich) was added, along with the cells, to trigger gelation. The CaCO_3_/GDL molar ratio was set at 0.125. The mixture was pipetted (20 μL) onto a Teflon plate, and hydrogel discs were casted between two plates separated by 750 μm-height spacers. After gelation (20 min) cell-laden hydrogel discs were transferred to pHEMA-treated (0.8 mg/cm^2^) 24-well culture plates. Thereafter, fresh medium was added and renewed after 1 h. 3D cultures of MCF10A were maintained in standard medium, which was changed every other day.

### Co-culture of MCF10A and hMF in the Hybrid 3D System

To create a co-culture hybrid system, MCF10A epithelial cells were combined with alginate and crosslinking agents, as described in section 3D Culture of MCF10A Epithelial Cells Inside RGD-Alginate Hydrogels, and the cell-laden gel-precursor solution was then used to fill the pores of the printed 3D scaffold (400 μM RGD) pre-seeded with hMF (after 10 days of mono-culture, with initial seeding density of 5.0 × 10^5^ cells/scaffold). Approximately 3.5 × 10^5^ epithelial cells were added per scaffold. After gelation for 30 min, the co-culture medium was added (70% v/v hMF media and 30% v/v MCF10A media), and hybrid 3D systems were maintained in co-culture for up to 14 days. As depicted in Supplementary Figure 1 culture of both cell types in the co-culture medium does not induce significant alterations on their metabolic activity, as compared to culture in their own media. After co-culture, the cellularized hybrid systems (whole-mounted samples) were analyzed by confocal microscopy after live/dead cells staining (section Metabolic Activity and Viability Assays) and immunostaining for different cellular and matrix components (section Whole-Mount Immunofluorescence Analysis by Confocal Microscopy). Retrieved cells were analyzed by flow cytometry (section Flow Cytometry Analysis of hMF and MCF10A Cells After Co-culture in the Hybrid System).

### Metabolic Activity and Viability Assays

Metabolic activity of hMF and MCF10A cells during monoculture was assessed using the resazurin assay. 3D constructs were incubated in 20% v/v resazurin (Sigma-Aldrich) solution in DMEM GlutaMax^TM^ for 2 h at 37°C. Thereafter, 200 μL/well were transferred into a 96-well black plate with clear bottom and fluorescence was measured (Ex=530 nm, Em= 590 nm) in a Synergy MxTM (BioTek) reader. For analysis of cell viability by the Live/Dead assay, 3D constructs were washed in non-supplemented DMEM GlutaMax^TM^ without phenol red, and subsequently incubated in this medium with 2.5 μg/mL of propidium iodide (PI) and 2.0 μg/mL of Calcein AM, for 45 min at 37°C. Then, 3D constructs were washed/maintained in fresh medium, and immediately imaged by confocal laser scanning microscopy (CLSM, Leica TCS SP5). Live cells stained in green (Calcein AM, Ex = 495 nm, Em = 515 nm), and dead cells stained in red (PI, Ex = 540 nm, Em = 615 nm).

### Cell Proliferation Assays

For total double-stranded DNA (dsDNA) and total protein quantification of gel-entrapped MCFD10A cells, hydrogels were dissolved with 0.25% trypsin/50 mM EDTA. Cells were recovered by centrifugation (1,500 rpm, 5 min), washed with PBS, centrifuged and stored at – 20°C until analyzed. Cells were lysed in 1% v/v Triton X-100 for 1 h at 250 rpm and 4°C. Samples were then diluted 1:10 in PBS and used for dsDNA quantification using the Quant-iT PicoGreen dsDNA kit (Molecular Probes, Invitrogen), according to manufacturer's instructions. Briefly, samples were transferred to a 96-well plate black with clear bottom and diluted in TE buffer (200 mMTris–HCl, 20 mM EDTA, pH 7.5). After adding the Quant-iT PicoGreen dsDNA reagent, samples were incubated for 5 min at RT in the dark, and fluorescence was quantified using a microplate reader with Ex/Em at 480/520 nm. Total protein in cell lysates was quantified by the BCA Protein Quantification Kit (ThermoFisher), according to the manufacturer instructions. In addition, cell proliferation was assessed by Ki-67 (Abcam, 1:100) immunostaining: MCF10A-laden hydrogels were fixed with 4 wt.% paraformaldehyde (PFA, Sigma) in HBSS (ThermoFisher) for 20 min, permeabilised for 5 min with 0.2% v/v Triton X-100/HBSS, and then incubated for 1 h in 2 wt.% bovine serum albumin (BSA) in HBSS to block unspecific binding. After incubation with primary antibody ON at 4°C, samples were incubated with goat anti-rabbit secondary antibody Alexa Fluor 488 (Molecular Probes, Invitrogen, 1:1000, 1 h, RT) and nuclei were counterstained with DAPI.

### Whole-Mount Immunofluorescence Analysis by Confocal Microscopy

To analyse the 3D printed scaffolds with hMF, the MCF10A-laden hydrogels and the combined hybrid system (co-culture), whole-mounted samples were fixed, permeabilised and blocked as described in section Cell Proliferation Assays. Then, immunostaining was carried out using the adequate antibodies for each specific analysis: to analyse cell morphology (actin cytoskeleton) samples were stained with flash phalloidin 488 (BioLengend, 1:100). To analyse ECM components, samples were incubated with the following primary antibodies (ON at 4°C): anti-rabbit Fibronectin (Sigma-Aldrich, 1:150), anti-rabbit Laminin (Sigma, 1:200), anti-rabbit Collagen Type I (Rockland, 1:100) and anti-mouse Collagen Type IV (Dako, 1:100). To analyse acinar-like structure formation, samples were stained with the epithelial-cells markers anti-rabbit E-cadherin (Cell Signaling Technology, 1:200) and anti-mouse β-catenin (BD Biosciences, 1:50), and also anti-mouse laminin antibody (Sigma-Aldrich, 1:50) to detect endogenous basal membrane formation. To detect hMF, anti-mouse Vimentin (Santa Cruz, 1:50) was used. In all cases, after incubation with primary antibodies, samples were incubated with goat anti-rabbit secondary antibody Alexa Fluor 594 or goat anti-mouse Alexa Fluor 488 (Molecular Probes, Invitrogen, 1:1,000, 1h at RT). Nuclei were counterstained with DAPI. Samples were mounted with VECTASHIELD® Antifade mounting medium (Vector Laboratories) and analyzed by CLSM.

### Flow Cytometry Analysis of hMF and MCF10A Cells After Co-culture in the Hybrid System

Fibroblasts and epithelial cells were analyzed after 2 weeks of co-culture by flow cytometry (FC). Cells were retrieved by incubating scaffolds in 0.25% trypsin/50 mM EDTA solution for 5 min for hydrogel dissolution and cell dissociation. Cells were centrifuged, fixed with 4% v/v PFA in PBS and then washed with FACS buffer (2% FBS/PBS). Fixed cells were blocked with 5 wt.% BSA in PBS (30 min) and stained with APC-conjugated anti-CD90 antibody (laser 647, dilution 1:100, BioLegend) for 45 min. After washing with FACS buffer, cells were permeabilised with 0.2% w/v Triton X-100/PBS for 5 min and stained with anti-rabbit E-cadherin antibody (Cell Signaling Technology, 1:200). After further washing, cells were stained with anti-rabbit Alexa Fluor 488 secondary antibody (for E-cadherin, dilution 1:1000) for 30 min, and finally washed and filtered. Cells were analyzed by FC using BD FACSCantoTM II (BD-BioSciences), with FACSDiva software (BD-Biosciences). All cells were kept on ice during processing and before FC. Results were analyzed using FlowJo v10.0 (FlowJo).

### Statistical Analyses

Statistical analyses were performed using GraphPad Prism 6.0 software version 6.01. To compare MCF10A DNA and metabolic activity, the non-parametric Kruskal–Wallis test was used. For protein analysis, parametric test ANOVA was used. For gene expression (CDH1 and Ocln), only E7 and E14 were compared using t-student test. Results for all analysis with *p*-value less than 0.05 were considered to indicate statistically significant differences (^*^p ≤ 0.05; ^**^p < 0.01; ^***^p < 0.001).

## Results

### Fabrication of 3D Printed Porous Scaffolds of RGD-Modified Alginate

To build the breast tissue model, a hybrid system was established, consisting of a 3D printed porous scaffold filled with an *in situ* forming soft hydrogel. RGD-modified alginate was selected for both components of the hybrid scaffold. The porous scaffold was fabricated by extrusion-based 3D printing as 12 × 12 mm^2^ crosshatch 6-layer structures, as shown in Figure 1A. The RGD-alginate solution was extruded though a nozzle into a CaCl_2_ bath, where filaments were crosslinked into hydrogels. Printing parameters, both related with the hydrogel formulation (alginate and CaCl_2_ concentration) and the extrusion process (rate, writing speed and needle height) were screened and optimized (data not shown). From all the tested printable formulations, the 4 wt.% alginate solution crosslinked in 12.5 mM CaCl_2_ was the condition yielding better shape-fidelity (Figures 1B–D), being thus selected for subsequent stages. 3D scaffolds were characterization in terms of porosity, surface area and shape-fidelity (Figure 1E). The printed scaffolds scored very high in all parameters (level 4 in porosity, 5 in all others), showing successful maintenance of shape, surface area and pore size.

### Building the Stromal Compartment: Fibroblast Culture in 3D Printed Scaffolds

**Figure 2:**
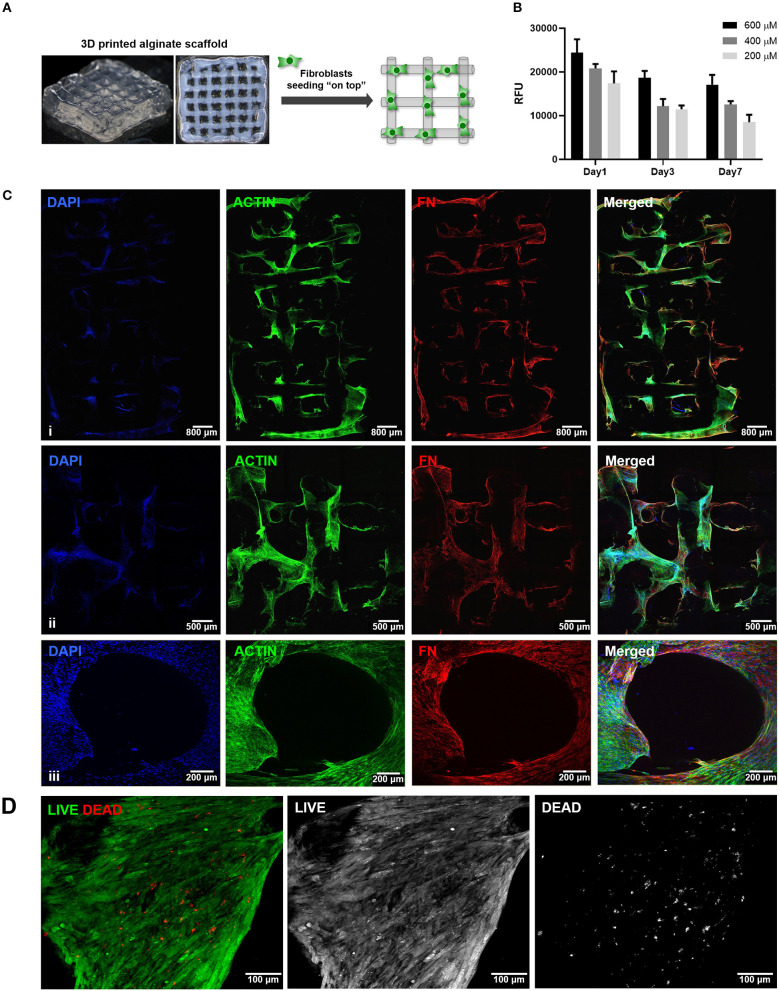
Seeding of hMF at the surface of 3D printed RGD-alginate scaffolds. **(A)** Schematic representation of the experimental strategy. **(B)** Metabolic activity of hMF seeded on alginate scaffolds of variable RGD amounts (200, 400, or 600 μM) (**C**i–iii) Representative CLSM images of 3D printed RGD-alginate scaffolds (5.0 × 10^5^ cells/scaffold, 400 μM RGD). Scale bars: 800 μm (i), 500 μm (ii), 200 μm (iii). (**C**iii) Zoomed-in image of a pore. All images show that hMF grown for 14 days on-top of printed scaffolds were able to spread (F-actin, green) and produce fibronectin-rich ECM (FN, red). **(D)** Live (green)/Dead (red) staining after 14 days of culture. Scale bars: 100 μm.

**Figure 3:**
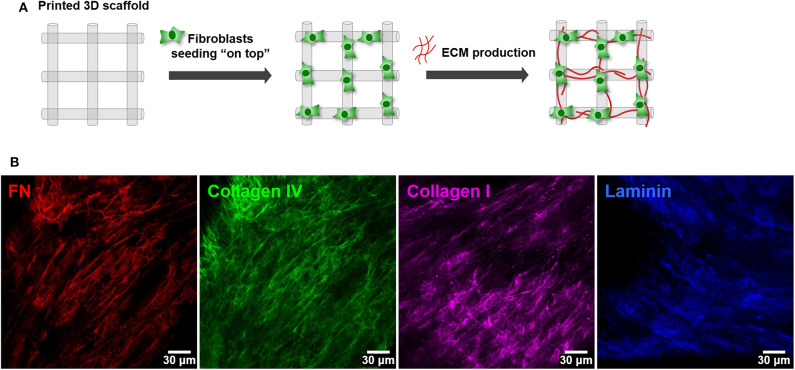
Deposition of hMF-derived ECM at the surface of 3D printed RGD-alginate scaffolds. **(A)** Schematic representation of the experimental strategy. **(B)** hMF produced endogenous ECM, rich in fibronectin (red), collagen type I (green), collagen type IV (pink) and laminin (blue), which were distributed throughout the scaffold's surface. Scale bars: 30 μm.

The second step consisted on the optimization of hMF seeding onto 3D printed scaffolds, to achieve uniform colonization and deposition of endogenous ECM at the surface (Figure 2A). Different parameters (RGD amount, cell density, suspension volume and seeding technique) were optimized. Unmodified alginate hydrogels are intrinsically non cell-adhesive, and thus chemical modification with RGD motifs was mandatory to promote integrin-mediated binding and cell adhesion. RGD concentrations ranging from 200 to 600 μM were tested, with 400 μM representing a good compromise, as it promoted sufficient cell adhesion at day 1 and supported metabolic activity throughout culture (Figure 2B). Different seeding volumes (from 10 to 50 μL) were screened for higher cell retention, and 30 μL shown to be ideal for filling the pores without excessive leakage. Seeding densities ranging from 1.0 × 10^5^ to 5.0 × 10^5^ cells/scaffold were tested, and cell spreading and scaffold colonization were analyzed for up to 21 days. A fraction of seeded cells was typically lost post-seeding, due to the high porosity of the scaffold, and therefore the highest density provided the best results. Under optimal hMF seeding conditions (5.0 × 10^5^ cells/scaffold, 400 μM RGD), the necessary time to achieve sufficient scaffold coverage was around 2 weeks. Samples were analyzed by CLSM and images were taken from both sides to evaluate if cells were able to uniformly colonize the whole scaffold (Figure 2C, increasing magnification from i to iii). After 2 weeks of culture, hMF were well-spread (f-actin staining, green), and presented a prototypical spindle shape (Supplementary Figure 1), forming a tightly packed layer at the scaffold surface, thus demonstrating good adhesion to the RGD-alginate hydrogel. They also presented high viability (Live/Dead assay (Figure 2D), with a large proportion of the cells staining in green. Moreover, hMF were able to assemble a fibronectin (FN) network, a key component of the ECM, which covered the whole scaffold surface, demonstrating homogenous distribution of secreted ECM components. Thereafter, a more detailed characterization with specific antibodies showed that the hMF-derived ECM was also rich in other key components, including collagen type I and type IV, as well as laminin (Figure 3). As depicted, these components assembled into organized fibrillar networks, and were not merely adsorbed at the scaffold.

### Building the Parenchymal Compartment: 3D Culture of Epithelial Cells Inside an *in situ* Forming Hydrogel

**Figure 4:**
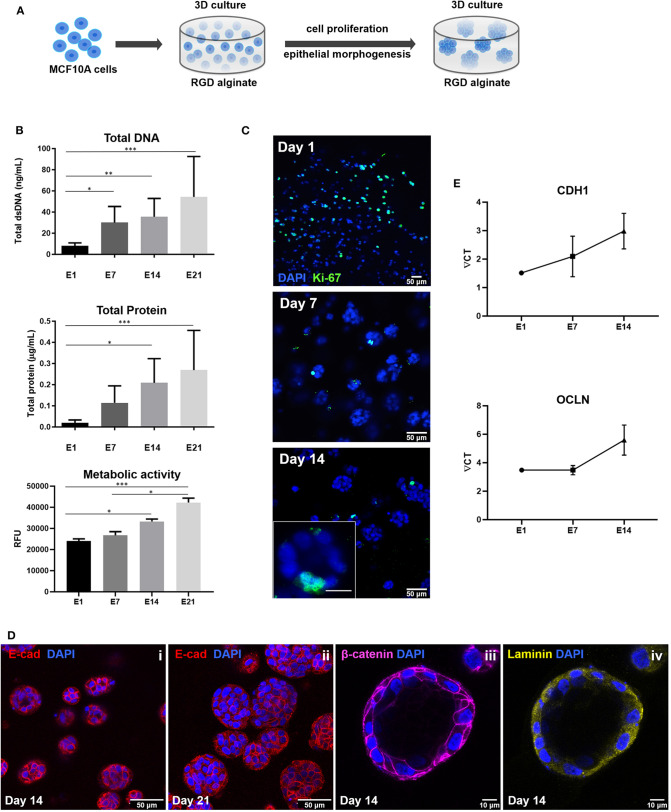
Establishment of MCF10A 3D cultures inside RGD-alginate hydrogels. **(A)** Schematic representation of the experimental strategy. **(B)** Cell proliferation estimated by total DNA (PicoGreen assay), total Protein (BCA assay) and metabolic activity quantification (Resazurin assay). **(C)** CLSM image of proliferative cells immunostained with the Ki67 nuclear marker. **(D)** CLSM image of epithelial spheroids, showing E-cadherin expression (i and ii, red) and formation of acini-like structures expressing basolateral β-catenin (iii, pink) and laminin (iv, yellow) at basal side. **(E)** RT-PCR analysis of CDH1 and OCLN mRNA expression. Data is presented as mean ± standard deviation. Statistical significance, *p ≤ 0.05; **p < 0.01; ***p < 0.001.

To build the parenchymal compartment, a previously optimized formulation of soft alginate hydrogels functionalized with cell-adhesion RGD peptides was used to simulate the 3D microenvironment of normal mammary gland (Bidarra et al., 2016). MCF10A epithelial cells were combined with a gel precursor solution of RGD-modified alginate (200 μM RGD) and ionic crosslinking agents, becoming entrapped in Ca-alginate hydrogels after gelation (Figure 4A). Under 3D culture conditions, MCF10A were able to proliferate, as demonstrated by the increase in DNA, total protein and metabolic activity along 3 weeks of culture (Figure 4B). This was corroborated by immunostaining of the proliferation marker Ki67 (Figure 4C). Cells grown as spheroids, which increased in size throughout culture time (Figure 4D). Cells in spheroids expressed E-cadherin, a prototypical epithelial marker, which was localized at the cell membrane, stabilizing cell-cell contacts (Figure 4Di). High levels of expression were detected for up to 21 days of culture, suggesting that cells maintained their epithelial phenotype (Figure 4Dii). Along time, some spheroids eventually lumenized, forming acini-like structures with peripheral nuclear alignment and apical-basal polarity, expressing basolateral β-catenin and assembling a laminin-rich basal layer (Figure 4Diii,iv). Maintenance of the epithelial phenotype was further confirmed by RT-PCR analysis of CDH1 (E-cadherin) and OCLN (occludin) expression (Figure 4E).

### Building the Co-culture 3D Model: Integration of Stromal and Parenchymal Compartments in a Hybrid System

**Figure 5:**
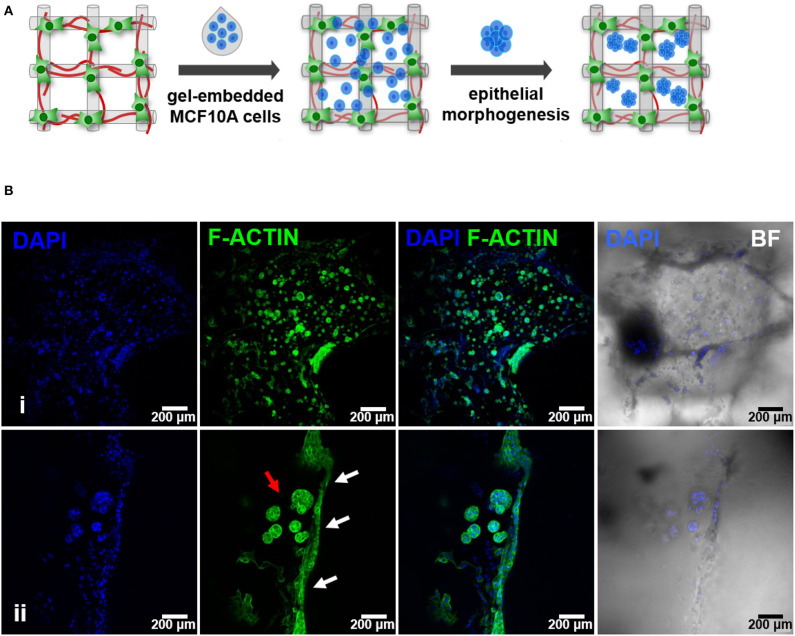
Hybrid system: hMF and MCF10A co-culture in 3D printed RGD-alginate scaffolds. **(A)** Schematic representation of the experimental strategy. **(B)** Spatial organization and morphogenesis of co-cultured hMF and MCFD10A cells within the scaffolds (F-actin in green, DAPI in blue). Scale bars: 200 μm (i) and 100 μm (ii).

**Figure 6:**
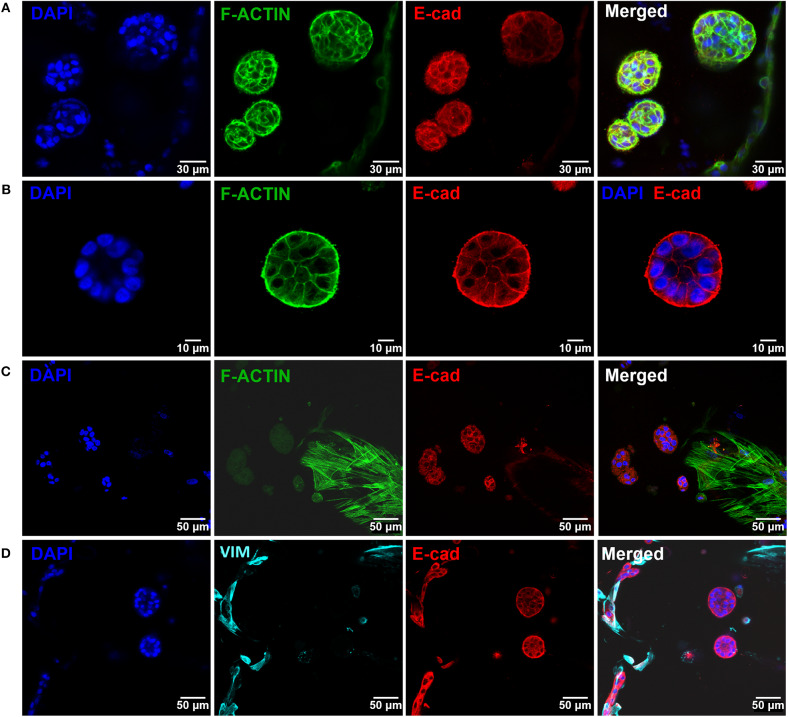
Epithelial morphogenesis of MCF10A epithelial cells in the hybrid system. **(A)** Epithelial cells formed spheroids in close proximity with fibroblasts. **(B)** Some spheroids matured into lumenized acini-like structures (images after 10 days of co-culture). **(C,D)** These structures were in close proximity with hMF. In all images: F-actin in green, DAPI in blue, E-cadherin in red, vimentin in cyan.

After establishing the stromal and epithelial compartments, these were integrated into a hybrid system for co-culture. Epithelial cells were combined with the RGD-alginate gel precursor solution and the mixture was added to cellularized (hMF) 3D printed scaffolds (pre-cultured for 2 weeks), forming a hydrogel *in situ*, inside the pores (Figure 5A). In the hybrid system, gel-entrapped MCF10A cells retained the ability to form spheroids, in close contact with previously seeded hMFs and their ECM (Figure 5B). After 2 weeks of co-culture, a large proportion of cells remained alive, showing high levels of calcein green staining (Supplementary Figure 3). Epithelial tissue-like morphogenesis in the hybrid system was further confirmed by the formation of lumenized acini-like structures after 10–14 days of culture. A more detailed analysis of such structures (Figures 6A–D), showed high E-cadherin expression, confirming the epithelial phenotype (Figure 6A). Some spheroids matured into acini-like structures, with well-defined basal-apical polarity (Figure 6B). Figures 6C,D allow to better discriminate between epithelial structures and fibroblasts, highlighting their close proximity.

### Retrieving Cells From the Hybrid System: Analysis of Cell Population After Culture

**Figure 7:**
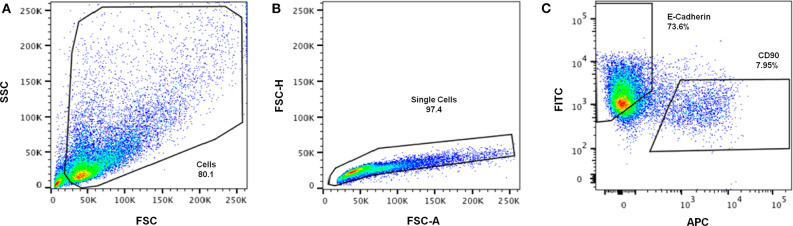
Representative gating strategy for immunophenotypic FC analysis of cells retrieved from the hybrid system. **(A)** Cells were first gated in a FSC-A vs. SSC-A plot, according to their size (SSC- side scatter) and granularity (FSC—forward scatter), **(B)** followed by debris and doublet exclusion on a FSC-A vs. FSC-H plot. **(C)** Dot plot of FITC (E-Cadherin) vs. APC (CD90) showing separation of epithelial-like and fibroblastic-like populations.

Cell retrieval after 2 weeks of co-culture was promoted by incubating the hybrid system in trypsin/EDTA solution, to revert ionic crosslinking and disrupt cell-cell and cell-matrix interactions. Cells were then analyzed by FC, using CD90 as fibroblastic marker and E-cadherin as epithelial marker (Figure 7). In Figure 7A, shows that it was that possible to isolate a viable cell population from the scaffold, with enough cells to be analyzed by FC. After excluding cell debris and duplets, to focus the analysis on single cells (Figure 7B), two distinct populations could be clearly separated as shown in Figure 7C. Fibroblasts stained positive for CD90, with around 7.95% expression, and MCF10A epithelial cells stained positive for E-cadherin, with expression leveling up to around 73.6%. Gates were selected based on the unstained control (Supplementary Information).

## Discussion

In the mammary gland, complex interactions between the epithelium and different other cell types direct the progression of normal mammary gland morphogenesis, being also implicated in pathological events (Dziegelewska and Gajewska, 2018). For instance, the interplay between the immune system and tumor progression is well-recognized, and a variety of pro- and anti-tumor immune cells can modulate the outcome toward tumor growth and progression or immune rejection (Zhu et al., 2019). Moreover, in breast tumors, signaling between cancer and stromal cells drives all stages of cancer initiation and progression, and tumor cells actively modify the composition of their stroma, which becomes significantly different from that of neighboring tissue (Hanahan and Weinberg, 2011; Balkwill et al., 2012). Thus, a better understanding of the crosstalk between mammary parenchymal (or tumor) cells and stromal cells is essential. However, *in vitro* platforms that conveniently model such complex interactions and reproduce dynamic signaling between epithelial and stromal cells are still lacking. To address this unmet need, this work focused on the development of a new 3D model of breast tissue, combining stromal and parenchymal compartments. In the hybrid system, gel-embedded mammary epithelial cells were incorporated in a 3D printed alginate scaffold, previously populated by mammary fibroblasts and their respective ECM. While this study focused on fibroblasts, which are abundant in breast stroma, other stromal cell types, such as adipocytes, may also be explored in future studies. It is well recognized that the specific signaling between adipocytes and breast cancer cells leads to phenotypical and functional changes of both cell types, influencing tumor progression (Wu et al., 2019).

To build multi-layered 3D printed scaffolds, several alginate formulations and printing strategies were tested. Alginate was selected as scaffolding material over other hydrogels due to its multiple advantages, namely because it presents: (i) tuneable rheological/gelling properties, allowing to achieve a good compromise between printability and mechanical properties of crosslinked constructs (Maia et al., 2014); (ii) intrinsic bio-inertness, coupled with presentation of functional groups for chemical modification (e.g., here RGD peptides were grafted to COOH groups to promote integrin-binding and adhesion, but grafting of other peptides, and combinations thereof, can be explored in future studies for additional control over cell response; Fonseca et al., 2011, 2013, 2014; Maia et al., 2014); (iii) transparency for routine monitoring of cell morphology and growth along culture, by optical microscopy; and (iv) reversible hydrogel formation by ionic crosslinking, allowing hydrogel dissolution with chelating agents for mild cell recovery after culture (Bidarra et al., 2014, 2016; Bidarra and Barrias, 2018). Additionally, alginate is a polysaccharide, not a protein, so cell-derived proteins and endogenous ECM produced during culture can be easily identified, as demonstrated herein. Extrusion printing was chosen over other printing strategies, like inkjet or laser printing, as it allows dispensing large hydrogel filaments, instead of droplets, which better suited our purposes. It also presents high deposition and printing speeds, improving scalability and serial scaffold production (Ozbolat and Hospodiuk, 2016). One possible limitation of extrusion printing is the resolution that in this case allowed a minimum feature size of around 210 μm (corresponding to the needle's internal diameter), which was nonetheless sufficient for the current application. After optimization of the process, we were able to fabricate 3D porous structures with sufficient shape-fidelity and reproducibility, according to the pre-specified architecture and porosity.

The second stage of this work was the establishment of the stromal compartment, where human mammary fibroblasts were seeded onto 3D printed porous scaffolds. Fibroblasts, together with adipocytes, are major cellular components of the mammary stroma, playing a key regulatory role in the mammary gland pathophysiology, not only by providing ECM-cues, but also by secreting bioactive compounds (Dziegelewska and Gajewska, 2018). Also, fibroblasts are the most abundant stromal cell type in epithelial tumors (Kalluri, 2016). The main aim was to achieve uniform colonization of the scaffold surface, without clogging the pores. As fibroblast are attachment-dependent cells and alginate is intrinsically non-cell adhesive, the polymer had to be chemically modified with integrin-binding RGD peptides (Rowley et al., 1999; Evangelista et al., 2007; Bidarra et al., 2010), which did not significantly affect the printing process. We tested different amounts of RGD and cell seeding densities, obtaining the best results with 400 μM RGD and 5 × 10^5^ cells *per* scaffold, which allowed fibroblasts to adhere, spread and populate the scaffold in its full depth. A static cell seeding approach was used, by simply dispensing the cell suspension on-top of the scaffold and letting the cells infiltrate the structure before adding culture media (Shi et al., 2018). Even though this method is known to induce very little damage to cells, it typically results in low cell attachment efficiency, due to cell leakage, especially in scaffolds with large pores, such as the ones used herein. Thus, dynamic seeding strategies involving the application of external forces, namely centrifugation or rotation, may eventually be a better option, and should be explored in future studies (Dar et al., 2002; Jaganathan et al., 2014; Chen et al., 2015). The metabolic activity of fibroblasts decreased from day 1 to day 4, which was mainly attributed to initial cell loss, and then attained nearly steady values. This was somehow expected, as in the mammary gland, fibroblasts are typically in a relatively quiescent state, proliferating slowly and remodeling their ECM (Dziegelewska and Gajewska, 2018). In our model, multiple protein components commonly found in native ECM were endogenously produced by seeded hMF, suggesting the establishment of an ECM-rich stromal niche. This was greatly facilitated by seeding cells on-top of hydrogel scaffolds, as such setting imposes no physical barriers for cell spreading and ECM deposition, in contrast to hydrogel-entrapment 3D cultures. In fact, preliminary studies with direct co-entrapment of hMF and MCF10A cells in RGD-alginate hydrogel were not successful, as fibroblasts remained essentially round, with scarce amounts of ECM components being detected, and only at pericellular regions (data not shown). Here, the well-defined stromal compartment is expected to subsequently contribute to epithelial cell signaling, influencing cellular activity (Sadlonova et al., 2004; Oskarsson, 2013).

The third stage of this work was the establishment of the epithelial compartment, where human mammary epithelial cells were embedded in a previously optimized RGD-alginate hydrogel formulation (Bidarra et al., 2016). We followed the same strategy reported for the entrapment and 3D culture of murine mammary epithelial cells, where best results had been obtained with alginate modified with 200 μM RGD, a density comparable to that of ECM-derived matrices (Huebsch et al., 2010), and stiffness around 200 Pa (as characterized in Bidarra et al., 2016), comparable to that of normal mammary tissue (Madsen and Cox, 2017). Since, MCF10A cells behaved as expected upon 3D culture in this hydrogel formulation, no further adjustments were needed. Entrapped MCF10A cells were able to proliferate, forming spheroids, most probably by clonal-growth as previously demonstrated (Bidarra et al., 2016). Some of these multicellular aggregates eventually maturated into prototypical mammary acini-like structure, with hollow central lumen. These structures presented typical features, such as apical-basal organization with segregation of polarization markers and deposition of a laminin-rich layer at basal side, which partially recapitulates the native basal lamina (Bidarra et al., 2016). The mRNA expression of typical markers (*CDH1, Ocln*) further confirmed the maintenance of an epithelial phenotype along 3D culture. The slight increase on the expression of these markers along culture may eventually be related with epithelial morphogenesis, as both codify cell-cell adhesion proteins that play key roles in maintaining tight junctions between cells in acini-like structures. Collectively, these results show that the previously optimized RGD-alginate hydrogels support normal morphogenesis of human epithelial MCF10A cells, similar to what had been observed for murine mammary epithelial cells (Bidarra et al., 2016).

Finally, the hybrid system was assembled, by combining hMF-seeded 3D printed scaffolds with hydrogel-entrapped MCF10A epithelial cells. The addition of ECM-producing fibroblasts to parenchymal cells in the 3D *in vitro* system was expected to promote not only heterotypic cell-cell, but also cell-ECM interactions, increasing similarities with *in vivo* systems. The gel-precursor solution with epithelial cells, efficiently filled the pores of the 3D printed scaffold, and the *in situ* formed hydrogel supported epithelial morphogenesis into spheroids and acini-like structures. These structures become in close contact with hFM and their ECM, allowing for paracrine signaling. Only a few hMF were able to migrate into the gel and infiltrate the epithelial compartment, because the designed hydrogel was soft and deformable (Bidarra et al., 2016), but most of them remained essentially attached to the surface of the printed scaffold, limiting the establishment of direct fibroblasts-epithelial interactions. In future studies, direct cell-cell interactions may be further promoted by tuning hydrogel composition. In particular, we could use protease-sensitive alginate, another derivative previously described by our group (Fonseca et al., 2011, 2013, 2014), which allow entrapped cells to remodel their pericellular space, supporting cellular activity/mobility inside the hydrogel, thus facilitating cell-cell interactions. Of note, by comparing a non-sensitive vs. protease-sensitive hydrogel, it will be possible to discriminate between the effect of “indirect” cell-cell signaling via secreted compounds (in the former) and “direct” cell-cell signaling (in the later). On the other hand, ECM production and cell-matrix crosstalk will certainly depend on the “activation” state of co-cocultured cells. In future studies, we intend to study the effect of fibroblasts activation (“normal” fibroblasts vs. cancer-associated fibroblasts, CAF) on epithelial acinar structures formations, and also test tumorigenic epithelial cells vs. normal ones, namely to understand the role of the ECM (and its alterations) on cell signaling.

When used as tools for investigating cell-cell and cell matrix crosstalk, co-culture 3D models should allow cell recovery under non-destructive conditions, for subsequent analysis of the different cell populations. We showed that our system could be dissolved with a chelating agent, to revert the ionic crosslinking, followed by enzymatic treatment with trypsin to disrupt cell-cell and cell-matrix interactions. Immunophenotypic analysis of cell populations by flow cytometry was performed, based on the expression of the fibroblastic/mesenchymal marker CD90 and the epithelial marker E-cadherin. The relative percentage of retrieved fibroblasts was low, which could be due to cell loss during seeding, lower proliferation rates during culture and/or loss along the isolation and/or staining process. Also, during 3D co-culture, cells may undergo gene/protein alterations, which might lead to variations in the expression of surface markers, and intermediate, double negative and double positive populations may appear. Thus, future studies should focus on improving the isolation/characterization of multiple sub-populations of cells after co-culture, namely by transcriptomics, to identify possible events, such as epithelial-to-mesenchymal transition (EMT), fibroblast activation and desmoplasia, among others.

## Conclusions

Collectively, our results demonstrate the successful establishment of a hybrid 3D model of breast tissue, with both stromal and parenchymal compartments, where epithelial cells and fibroblasts are co-cultured in the same 3D microenvironment. Cells may communicate by paracrine signaling, as cell-secreted compounds may easily diffuse through the highly permeable hydrogel. Modification of the hydrogels with protease-sensitive motifs may further enhance cellular crosstalk and direct cell-to-cell signaling. By partially recreating a tissue-like environment, namely by facilitating heterotypic interactions between stromal and epithelial cells, our 3D model is expected to provide a useful *in vitro* platform to study the dynamics of breast tissue alterations, both under healthy and pathological conditions. In particular, future studies should focus in the identification of mechanism underlying the role of epithelial-stromal crosstalk in breast tumor formation/progression, using complementary techniques.

## Data Availability Statement

The raw data supporting the conclusions of this article will be made available by the authors, without undue reservation, to any qualified researcher.

## Author Contributions

PB and MC designed experiments, acquired and analyzed the data and wrote the manuscript. SB helped in the acquisition and analysis of data related to epithelial 3D cell culture. SN helped in the development and optimization of scaffolds 3D printing. CB designed and supervised the whole work, analyzed the data and wrote/revised the manuscript.

## Conflict of Interest

The authors declare that the research was conducted in the absence of any commercial or financial relationships that could be construed as a potential conflict of interest.
