# Supplementary material for: Reshaping in vitro Models of Breast Tissue: Integration of Stromal and Parenchymal Compartments in 3D Printed Hydrogels
Source: Front Bioeng Biotechnol. 2020 Jun 11;8:494. doi: 10.3389/fbioe.2020.00494 (PMC7300215; doi:10.3389/fbioe.2020.00494)
Supplement: Supplementary file 1 [file Table_1.DOCX]

Supplementary Material

# Supplementary Figures


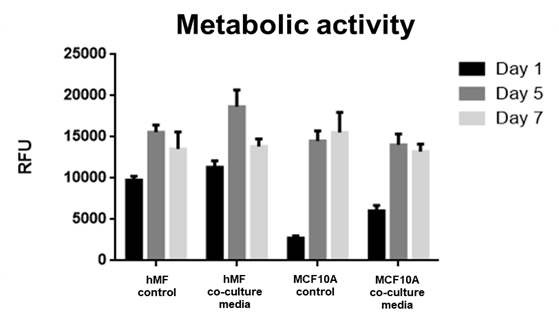


**Supplementary Figure 1.** Metabolic activity (resazurin assay) of hMF and MCF10A cells after 1, 5 and 7 days of culture in their own media (control) and in co-culture medium.


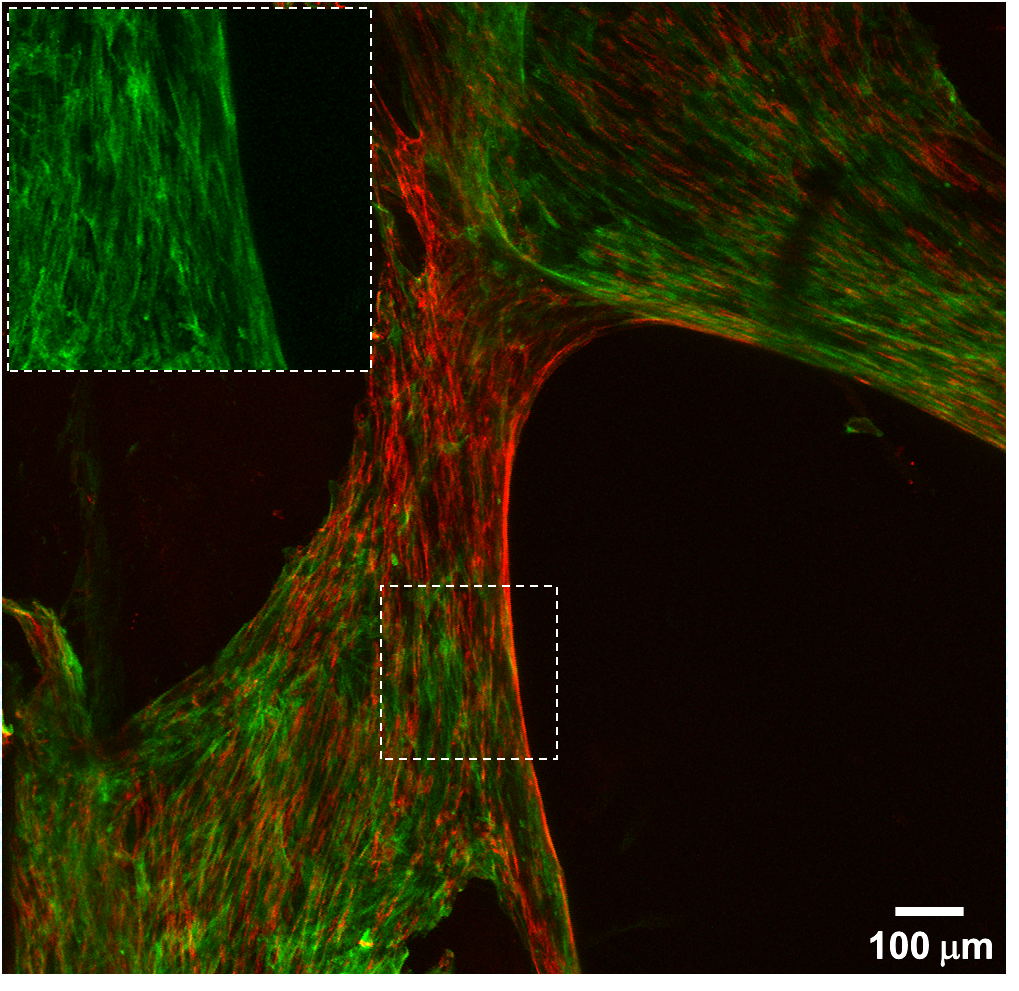


**Supplementary Figure 2.** Detail of hMF (F-actin, green) after 14 days of being seeded on 3D printed RGD-alginate scaffolds, showing that fibroblasts were well spread and presented a prototypical spindle shape, forming a tightly packed layer at the scaffold surface, thus demonstrating good adhesion to the RGD-alginate hydrogel. The image also shows fibrillar filaments of endogenously produced fibronectin (FN, red), showing that fibroblasts were actively producing their own ECM.


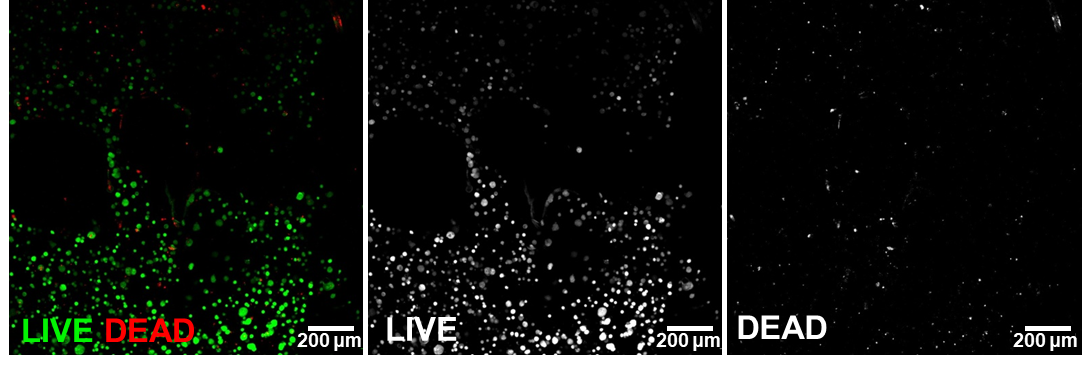


**Supplementary Figure 3.** Live (green)/Dead (red) staining of hMF and MCF10A in 3D printed RGD-alginate scaffolds after 14 days of co-culture. Scale bar: 200 µm.


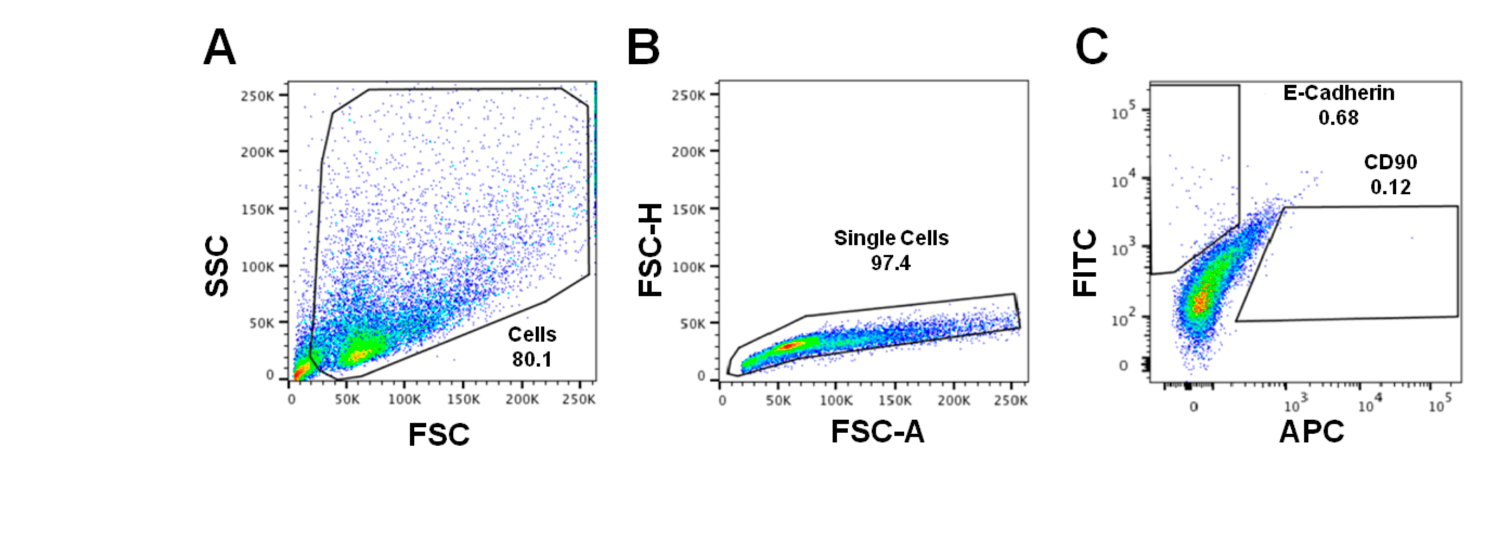


**Supplementary Figure 4.** Back gating for flow cytometry analysis of fibroblastic and epithelial cells after being retrieved from the hybrid system. **A)** Light-scattering profile of cell population isolated from the whole sample according to their size (SSC- side scatter) and granularity (FSC – forward scatter). **B)** Isolation of duplets and cell debris, single cells analysis, basis on height and area (FSH-H and FSC-A, respectively). **C)** Dot plot of FITC (E-cadherin) versus APC (CD90) showing non-significant auto-fluorescence of unstained sample.
